# Co-Designing Research for Sustainable Food Systems and Diets with Aboriginal Communities: A Study Protocol

**DOI:** 10.3390/ijerph21030298

**Published:** 2024-03-04

**Authors:** Carla Vanessa Alves Lopes, Seema Mihrshahi, John Hunter, Rimante Ronto, Renee Cawthorne

**Affiliations:** 1 Department of Health Sciences, Macquarie University, Sydney 2109, Australia; 2 Murama Cultural and Healing Centre, Sydney 2127, Australia; 3 Botanic Gardens of Sydney, Sydney 2000, Australia

**Keywords:** co-design, Aboriginal, Indigenous knowledge and methodologies, Indigenous food systems, native food, nutrition, sustainability, health promotion

## Abstract

(1) Background: Food choices and systems have contributed to various health and environmental issues, resulting in the global syndemic (obesity, undernutrition and climate change). Studies show that revitalizing Indigenous food systems and including native plant-based foods in our diet may be important for promoting health, reducing diet-linked chronic diseases and mitigating environmental changes. However, it is still a challenge to ‘Indigenize’ research by including Aboriginal people in all project phases to achieve culturally appropriate collaboration. We describe the development of a protocol using co-design methods to explore how knowledge can be exchanged around Aboriginal food practices related to native plant-based foods to facilitate benefits and share opportunities for sustainable food systems and Aboriginal aspirations, as well as to promote health in these communities. (2) Methods: This qualitative study includes five phases. In Phase I, we will start building a trusting relationship with the communities and train the research team. In Phase II, we will receive the consent to co-design and establish the Aboriginal Reference Group to ensure that Aboriginal people will lead this project. In Phase III, the investigators will run the interviews/focus groups and record the discussions about the community’s place-based needs, understanding the community aspirations for a sustainable food system and the potential opportunities for doing research and strengthening community between research and community. In Phase IV, the records will be analyzed with the Aboriginal Reference Group, and summaries will be shared with community members. Phase V will establish case studies to support the implementation of community aspirations. (3) Discussion: This study protocol describes the process of ensuring that research for sustainable food systems meets Indigenous aspirations and health promotion in Indigenous communities.

## 1. Introduction

The current global food system is unsustainable from a natural resource perspective, and is one of the main contributors to the global syndemic of obesity, undernutrition and climate change [1,2,3,4]. Results from the latest Australian National Health Survey [5] showed that 1.2 million Australians (4.9%) have diabetes. Over 65% of Australian adults are overweight or obese, with 47% of people suffering from one or more chronic conditions. The report also showed that only 7.5% of adults met the guidelines for the recommended daily intake of vegetables (5–6 or more servings daily), and 51.3% met the guidelines for fruit recommendations (2 or more servings daily) [5]. Moreover, in the last decade, extreme weather events such as drought, floods and bushfires have increased, resulting in severe consequences to Australia’s environment and human health [6,7]. The effects of these events on Aboriginal communities are further exacerbated. Aboriginal people have a reduced life expectancy compared to non-Aboriginal people [8], the prevalence of obesity among Aboriginal people is more than 15% higher than non-Aboriginal people [9], the rate of type 2 diabetes is 3.3 times higher than non-Indigenous adults and one in four Indigenous Australians experience food insecurity [10]. The effects of climate change also impact Aboriginal communities disproportionally due to their close and dependent relationship to nature. The depletion of natural resources, socioeconomic disadvantage and higher rates of climate-sensitive health conditions have resulted in large health gaps [11,12]. The concurrent contribution of poor diets due to affordability and accessibility issues [10] as well as the intergenerational transmission of stress and trauma due to colonization [13,14] makes the gap even wider.

To mitigate the problems associated with this and achieve the sustainable development goals, international organizations and experts recommend increasing plant-based food consumption due to its lesser impact on the environment coupled with the associated health benefits [15,16]. Studies also report that revitalizing Indigenous food systems is crucial to face the global syndemic [17,18,19]. Indigenous people know native species such as fruits and vegetables that are abundant in nature, counting 1097 edible species worldwide, which can contribute to a more diverse diet and can adapt to difficult environmental conditions such as poor-quality soil and cold/hot weather. Moreover, these species have demonstrated good resistance to pests and diseases [17]. Traditional plant-based foods also have health benefits such as antihypertensive, antihyperglycemic and antidyslipidemic properties, making them an excellent tool for combating diet-linked chronic diseases [18]. Our recent review showed the social, environmental and economic benefits of Australian native plant-based foods, which have the potential to make our food system more equitable, resilient and sustainable [20]. Australian native plant-based foods are nutrient-rich, have bioactive compounds linked to chronic disease prevention (such as diabetes and cancer), present a cultural identification for Aboriginal people and have environmental stress tolerance and some positive impacts on the ecosystem [20].

Nevertheless, European and British invasions between the 18th and 19th centuries devastated traditional food practices and culture for Aboriginal people. This colonization also supported racist laws and policies and introduced Western ingredients into Aboriginal diets, such as replacing traditional foods with foods rich in calories, simple sugar and saturated fats [21,22]. Colonization is also traumatic for Indigenous people due to the positivist research paradigm imposed by Western researchers, in which the methods negate Indigenous experiences, with no benefits for communities [23,24,25]. In the last decades, Indigenous people have advocated for more inclusive and participatory research methods where Indigenous communities can guide the research according to their needs (in their words, “no more research about us without us”) [19]. In 2007, the United Nations’ declaration on the rights of Indigenous peoples also highlighted the importance of having research on Indigenous people governed by Indigenous people [26]. Kovach [25] states that Indigenous methodologies must be participative in that all research phases apply the Indigenous lens with respect, reciprocity, relevance and responsibility.

Participatory research is an umbrella term that includes approaches that engage communities and stakeholders, recognizing their values and knowledge [27]. In 1970, the Brazilian educator and philosopher Paulo Freire, a pioneer and leading advocate of critical pedagogy, stated that individuals can determine their own needs and improve their lives in his book *Pedagogy of the Oppressed* [27,28]. International organizations such as the United Nations Education, Scientific and Cultural Organization (UNESCO) used Freire’s ideas to work with marginalized communities with a participatory approach to support policy changes [27].

Community-based participatory research (CBPR) is one of the methods used to equally incorporate researchers and community members, recognizing their potential and needs with respect and reciprocity [29,30]. Co-design or co-producing research is a collaborative approach that can be used in CBPR, where researchers and communities work together with a horizontal relationship, sharing power and responsibilities through all steps of the project [31,32]. The key principles of a co-design research project are as follows: building and maintaining trusting relationships, which is essential to unlock people’s potential that enables co-producing to happen; sharing power, which facilitates community engagement and establishes a research reference group; ensuring the inclusion of all perspectives and experiences; respecting and valuing the knowledge from all equally; and working with reciprocity, where everyone benefits from the research [32].

Considering the underrepresentation of Aboriginal voices in food policies [33] and the social, environmental and economic benefits of Aboriginal food practices and Australian native plant-based foods for sustainable food systems [20], co-design research projects with Aboriginal communities may play a crucial role in including Aboriginal aspirations in food policies and achieving more resilient and sustainable food systems. This paper proposes a protocol for community involvement and co-design to explore how knowledge can be exchanged around Aboriginal food practices to facilitate benefits and sharing opportunities for sustainable food systems and Aboriginal aspirations in two different urban Aboriginal communities in Sydney. This community-based participatory research will apply a qualitative approach in its first phase, using interviews and focus groups to explore the communities’ needs, aspirations and experiences with Australian native plant-based foods. The second approach will be a quasi-experimental study where we will document the community project related to Australian native plant-based foods. In this paper we will use ‘Indigenous’ to collectively refer to associations related to both Aboriginal and Torres Strait Islander Australians. The term ‘First Nations’ will relate to the diversity of distinct pre-colonial Indigenous countries with their own independent languages and identities geographically situated across the continent. Additionally, the term ‘Aboriginal’ generally relates to all First Nations of mainland Australia and Tasmania [34].

## 2. Materials and Methods

This qualitative study uses a co-design approach utilizing focus groups and individual structured interviews with a “yarning” communication style. Focus groups are used to extract data resulting from participants discussions and give richer information about different experiences and perspectives [35]. Yarning circles are an appropriate method for community-based research with Indigenous Australians that involves storytelling, sharing knowledge and actively listening to enable deep and meaningful conversations [36]. Yarning circles allow Indigenous communities to participate in the research decision-making, becoming an active voice for their concerns and community’s needs [36]. The project’s design is based on an Indigenous methodology to ensure Indigenous leadership, governance and community-based engagement occurs.

### 2.1. Community Settings

The study will work with two Aboriginal community managed sites in Greater Western Sydney, New South Wales. Community A, Murama Healing Space, is located at Sydney Olympic Park. It is a place for local, regional and international Indigenous art, learning and collaboration. The space has an Aboriginal community garden and an interpretive cultural education trail titled the Wangal Walk. This initiative is a new way for the government and community to work together. The space has a considerable collection of artwork, stories and cultural materials from the community. Community B, Baabayn Aboriginal Corporation, is located in Mt. Druitt and is linked to Community A through a government partnership which jointly contributes to the cultural needs of the Indigenous community of Greater Western Sydney. Baabayn was founded by five Aboriginal Elders and aims to provide a place of healing where Aboriginal people can connect with their culture, form a strong sense of belonging, recover from past traumas, regain their self-esteem, and realize their potential. Baabayn supports thousands of Aboriginal individuals and their families living in the local area. The organization its nestled between Blacktown and Penrith, two of the largest inter-tribal urban Aboriginal communities in Australia with around 22,737 people [37,38]. Baabayn also has a community garden where members of the Aboriginal community can grow native foods.

Both Murama and Baabayn strongly desire to revitalize the knowledge of traditional food systems around native plant-based foods and recently started growing species such as Yam Daisy (*Microseris lanceolata*), Native Lemongrass (*Cymbopogon ambiguous*), Warrigal Greens (*Tetragonia tetragonioides*), Finger Lime (*Citrus australasica*) and Lemon Myrtle (*Backhousia citriodora*). The first author has supported the community gardens, planting and watering the plants as part of the relationship-building process.

### 2.2. Methods

**Figure 1:**
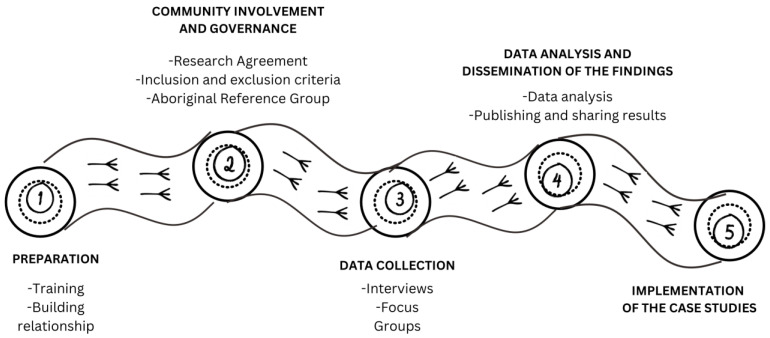
Five phases of the co-design protocol.

The current co-design protocol will be undertaken in five phases. Figure 1 describes the five phases of the co-design protocol: (1) preparation, (2) community involvement and governance, (3) data collection, (4) data analysis and dissemination of the findings, and (5) implementation. However, in line with learning from Indigenous methodology, many of the processes and steps may change according to the lived experience during the research process and community suggestions.

The project is expected to run for three years. In the first year, we will run phases I and II ((1) preparation and (2) community involvement and governance). Building a relationship with communities is an ongoing process and will continue throughout the project and we hope long after. Year two will be for phases 3 and 4 ((3) data collection and (4) analysis and dissemination of the findings). Phase 5, implementing the case studies, will run in the last year.

#### 2.2.1. Phase I: Preparation

##### Training/Developing Research Team Cultural Competency

Integrating the diverse knowledge of our research team is a precondition for effective teamwork in the current project. CL has a nutrition science and public health background, and has participated in some environmental and public health research groups. JH is the leading academic Indigenous researcher, and his work focuses on community-based solutions that facilitate self-directed, sovereign change as a means to address marginalization and disadvantage affecting Aboriginal communities. SM has expertise in public health and community-based approaches for improving food security in vulnerable groups. RR has expertise in public health nutrition and sustainable healthy diets.

Considering the fact that not all researchers are Aboriginal, this phase involves the research team undergoing training to build their capacity to work with Aboriginal communities with respect and reciprocity. The Manawari (Dharug language meaning “discover new knowledge”) staff Aboriginal cultural safety training will be delivered by staff at Walanga Muru, Macquarie University [39]. The training aims to prepare the university staff to work with Aboriginal communities to develop a meaningful understanding and respect for Aboriginal cultural values, beliefs, practices, histories and knowledge. The training has three phases: I, pre-workshop online modules; II, face-to-face workshops and; III, survey/evaluation. The training provides materials and discussions, including how to use appropriate Indigenous terminologies, Aboriginal history, kinship, identity, the impact of invasion/colonization, and how to work with respect, responsibility and reciprocity in Aboriginal communities. After all these stages are completed, participants will receive a certificate from the university [39].

##### Building a Trusting Relationship

A trusting relationship with the communities will be fostered as an essential step for the communities to analyze whether the research team is welcome and meets to their expectations. In this phase, the relationship will focus on aspects of life where the researchers can share their life stories, dreams, aspirations and concerns, and can learn about the community in a reciprocal way that sees experiences and knowledge being exchanged. The research team will attend the planned activities by the communities, being involved in their routine. The Aboriginal chief investigator, who has kinship connections with First Nations peoples of NSW and has links to the communities, will facilitate the building of this relationship with the two primary case study communities. There will not be a fixed number of visits or a specific period for community visits because it will depend on the trusting relationship built.

#### 2.2.2. Phase II: Community Involvement and Governance

##### Letter of Support and Research Agreement

The Aboriginal chief investigator will invite prospective communities to participate in the co-design process. Interested communities will present a letter of support, formally acknowledging their wish to be part of the project, recognizing common goals in land-based education, revitalizing cultural practices and healing and supporting the research activities by facilitating community engagement with their stakeholders. Then, a research agreement will be developed to ensure integrity, benefits, respect and cultural safety during the research project. The research agreement will contain the research aims and methods, benefits to the Aboriginal communities, researchers and participating organizations and communities’ commitment, ownership of copyright and intellectual property management, conflict resolution, complaint processes and funding details.

##### Inclusion and Exclusion Criteria

People who identify as Aboriginal aged 18 years and above will be eligible to participate in the co-design focus groups/interviews that will take place in the Aboriginal communities. All genders and ages above 18 years can be included, and all community members will be eligible. However, community members in leadership positions such as community directors, elders and members involved in the garden’s projects will be preferable as they can represent the community’s needs and aspirations for sustainable food systems. Aboriginal community members reflect any Indigenous people accessing the services offered by Baabayn and Murama in the local area. This includes individuals and their families that use the variety of Baabayn and Murama culture and wellbeing support programs. Participants may include knowledge holders and healers, alongside general community members.

##### Aboriginal Reference Group

An Aboriginal Reference Group (ARG) will be organized to participate in the research process. The role of the ARG is to guarantee that the project will be designed and driven with a commitment to Indigenous self-leadership of research, Indigenous people’s rights, sovereignty and aspirations. The terms of reference will be developed, and it will reflect the scope of the Aboriginal Reference Group, members’ names and Aboriginal background, members’ agreement, and meeting structure. The roles and responsibilities of the ARG are to guide the project’s design and implementation in a culturally safe way and provide advice and guidance on appropriate approaches and content for the focus groups, workshops, papers, conferences and interview guides. The ARG will also provide input for the research project regarding needs and aspirations from the Aboriginal communities, provide advice and input on strategies to support better engagement to community members, discuss issues regarding the research project to solve it for the community’s benefit, guide the research team in deciding how community members will participate in data collection and analysis and how the results will be shared with the community and public.

The ARG will have representatives from the two Aboriginal communities studied. We expect a total of 4–6 ARG members from both communities, however, the number of participants will depend on the community’s interest. All community members will be invited to join the group. Interested community members will be invited for an initial 12-month term, which can be extended according to the member’s interests. Membership will cease if the members decide to leave the group or when the agreed term of appointment expires. The members will receive a $50 voucher for participating in each meeting. All the ARG members will be invited to attend meetings, review agendas and documents prior to meetings, suggest agenda topics to be discussed, and allow members to give their opinions, maintaining confidentiality. The group will operate until the project is finalized, and members will nominate an agenda to be discussed. The meetings will have a duration of one to two hours, and only members or invited guests will be allowed to participate. The members will meet at the Murama Healing Space or Baabayn Corporation at a day and time to be decided by the team, and a minimum of 51% of the members are required for a meeting to proceed.

##### Ethics Approval

This study has been approved by the Macquarie University Human Research Ethics Committee [Project ID 12478] on 13 December 2022 and by the Aboriginal Health and Medical Research Council of NSW ethics [Project ID 2164/23] on 3 October 2023. We will conduct the research in accordance with the National Health and Medical Research Council (NHMRC) guidelines for ethical research with Aboriginal people [40].

#### 2.2.3. Phase III: Data Collection

##### Participant Recruitment and Consent

The Aboriginal chief investigator with the research team will approach the community members in person to explain the study objectives. Those interested in participating will be invited (via email) to a face-to-face meeting where the method and consent form will be explained. Participants will be allowed to withdraw from the focus group or individual interview at any time without any consequences. As a form of reimbursement, we will offer a voucher worth AUD 50 for each participant. We expect to interview 10–15 community members, however, the number of participants will depend on members’ interests and data saturation from the focus groups and interviews.

##### Structure of Focus Groups and Interviews

Focus groups with the yarning communication style and individual interviews will be used to understand the community’s place-based needs and aspirations for a sustainable food system, and to discuss potential opportunities between research and community as well as co-design the project. To facilitate the members’ participation, each focus group will range from 6 to 10 people, and the number of the focus group can be different according to the number of participants. The focus group and interviews will be in English (as all community members can speak English) and will take approximately 45–60 min. We will break down the focus groups into two or three parts if necessary. Individual interviews will be offered to participants as an alternative to focus groups. If participants cannot attend in person, there will be an opportunity to participate in a virtual interview or focus group using Microsoft Teams. We will use semi-structured questionnaires which will be elaborated with and approved by the ARG to conduct the interviews and focus groups. The ARG will also guide the research team about their communities’ local customs and traditions to open the focus groups, such as with an Aboriginal Elder. The consent form will be explained, and the research team will clarify all questions that are raised. Definitions of sustainable food systems will be explained to all participants using the concept and framework for sustainable food system by the Food and Agriculture Organization of the United Nations [41]. Then, participants will be encouraged to share stories and experiences. They will also answer questions about the community’s needs for more sustainable food systems, their experiences with Aboriginal food practices and Australian native plant-based foods and the best ways to co-design a collaborative research project to contribute to a meaningful transformation of food systems through Aboriginal aspirations. Indigenous knowledge mobilization is defined as the sharing of culturally relevant and valuable health information and practices led by Indigenous people to improve Indigenous health status, programs, services and policies [42]. In the context of this research project, Indigenous knowledge mobilization emerged from the shared stories, experiences and aspirations of the community, and is a way to improve our food system to a more sustainable, equitable and culturally appropriate one and improve Indigenous food security and health.

##### Documenting the Focus Groups and Interviews

Discussions during the co-design focus groups and interviews will be recorded and transcribed verbatim. Also, research team members will take notes from the discussions. Participant identities will be hidden in order to protect their privacy and confidentiality. The transcripts will be checked for accuracy and sent to each participant to review, ensuring that they reflect what they intended.

#### 2.2.4. Phase IV: Data Analysis and Dissemination of Results

##### Data Analysis

The qualitative data will be imported into NVivo software version 20, where thematic data analysis will be applied and carried out by the research team and members of the ARG, following best practices for qualitative methods. Thematic analysis is a method that identifies codes and themes in qualitative data to communicate the analyst’s story of the data [43]. An inductive analysis will be conducted where codes and themes are generated from familiarizing the data [43]. We will follow the five phases of qualitative data analysis suggested by Terry et al. [43]: (1) familiarizing ourselves with the data, (2) generating codes, (3) constructing themes, (4) reviewing the potential themes, and (5) defining and naming themes and producing/presenting the report. According to Troman and Jeffrey [44], the data analysis must be reciprocal in a cross-cultural project, where the team must find ways of discussing and analyzing the data together. Wasser and Bresler [45] call this an interpretive zone, where the research team and participants can interpret the data and share field notes, videotapes and memos for group discussion. Considering this, the research team will discuss with the ARG the best ways to analyze the data during and after the data collection to review the findings, identify outstanding quotes and write a summary for the future presentation of results [46].

##### Dissemination of Results

Working in collaboration with the ARG, the findings of the co-design study will be disseminated to the local Aboriginal community through emails, community meetings, yarning circles and printed research summaries. Community members will be supported so as to provide feedback and suggestions (if necessary) to ensure that the research team’s interpretations are aligned with their views. We will acknowledge the sources of information and all who have contributed to the research project through acknowledgements in resulting publications, workshops, conferences and other events. Results will be presented at national and international conferences and published in peer-reviewed journals. The ARG members will be invited to be co-authors of the publications and presentations.

Findings from this study will also be applied to develop booklets with the main information from this collaborative research, such as photos of plants/foods, techniques, history, recipes and nutritional information that the community can use in its routine. This will include online, pdf and video resources that share both information and stories.

#### 2.2.5. Phase V: Implementation of the Case Studies

Phase V will establish case studies to support the implementation of community aspirations with a set of identified objectives (according to the collected data in Phase III) and for knowledge mobilization based on the findings. The case studies are collaborative projects identified through consultation with Baabayn and Murama voicing the aspirations of the local Aboriginal communities. The case studies will be the development and implementation of the booklets and other materials, revitalizing the Aboriginal knowledge and native food knowledge that the community can use in its routine.

## 3. Discussion

This study protocol focuses on creating a method for community engagement that is culturally safe, acceptable, adaptable and meets the needs of the community. The co-design method is the first step of this broad project, however co-production and the participatory approach will continue throughout all of stages of the project.

Anderson et al. [47] highlight the importance of aligning the co-design process with First Nation culture and values. In their study, the authors developed a set of key principles and best practices for co-design with First Nations Australians. These principles include First Nations leadership, a culturally grounded approach, respect, a benefit to the community, inclusive partnerships, transparency and evaluation. Studies have shown the importance of co-design research in food security projects in Indigenous communities [48,49]. Richmond and Dokis [50] interviewed program leaders, volunteers and recipients to narrate the creation and daily operations of an Indigenous food bank in Canada. The program offered Indigenous people living in the city the possibility of accessing fruits, vegetables and traditional foods, where traditional foods are highly inaccessible due to their cost, seasonality and specialized knowledge required for their preparation. The participants highlighted the relevance of restoring traditional food systems and cultural connections through land-based learning, growing traditional foods and food sharing. This community-led research empowered Indigenous people and provided an opportunity to meet health and nutritional needs. However, including Aboriginal aspirations and Indigenous methodologies in nutrition research and in food policies for more equitable, resilient and culturally appropriate food system is still a challenge [33,51]. This protocol represents an essential first step to Indigenize sustainable food systems research, which is also an essential strategy to close the health and nutritional gap between Indigenous and non-Indigenous populations.

Participatory research using co-design and yarning resources with Indigenous communities is well described in the literature as a legitimate research method and culturally safe approach for local Aboriginal communities [52,53,54]. The topic of sustainability in Aboriginal food systems is complex and involves different social, economic and environmental impacts and challenges [20]. Therefore, co-design is necessary due to its potential to focus on the process and results, respecting the needs of the community, having a power-sharing process, building the community’s strengths and capacities as well as focusing on the relevant local issues to support the public policies. Co-design requires enough time to build a trusting relationship for yarning, sharing experiences and stories when different knowledge is valued. Successful co-design projects need support and conditions for inclusivity [55]. Even though studies with a participatory approach are challenging, labor-intensive and time-consuming [23], Ruwhiu et al. [56] state that transformative solutions that can drive broader social changes emerge at the local level.

Studies have shown the CBPR approach’s impact on promoting health in different populations and settings [57,58]. Lindsjo et al. [57] used a CBPR to identify conditions for health promotion with women migrants in a socially disadvantaged area in Sweden. The authors showed that the participants, being involved in the research process from the start, were empowered to discuss their resources to promote their health and what they wanted to learn about the healthcare system. Cristiani et al. [58] conducted qualitative research using the CBPR approach. They showed that the method was essential for getting feedback from students and staff, which was helpful in the implementation of strategies to improve healthy lifestyles among students in a community school [58].

This study can benefit the communities in land-based education, revitalization of cultural practices related to native foods as well as cultural healing, which can positively impact their health and wellbeing. CBPR has excellent potential for health promotion among socially disadvantaged groups.

This study can promote health at the individual and local level, revitalizing their knowledge about traditional foods they can use to improve their health and the environment, improving the quality of life and promoting the health of Aboriginal and non-Aboriginal people.

### Limitations

The main limitation of this protocol, which is present in many other participatory studies, is the lack of precise details such as interview guides, the exact structure of the focus groups and the case studies as they will be refined by the participants as the project progresses. Enablers, barriers and strengths will be known to the research team after the co-design process, which will be transparently evaluated and documented with the help of advice by the ARG. Despite these limitations, we can ensure that the main benefit of this study is giving more voice and visibility to Aboriginal aspirations in food systems research.

## Data Availability

The data used during the current study is available from the corresponding author upon reasonable request.

## Abbreviations

UNESCO—The United Nations Educational, Scientific and Cultural Organization; CBPR—Community-Based Participatory Research; ARG—Aboriginal Reference Group; AH&MRC—Aboriginal Health and Medical Research Council of NSW; NHMRC—National Health and Medical Research Council.
